# Electrosprayed Magnetic Poly(butyl methacrylate-*co*-(2-dimethylaminoethyl) methacrylate-*co*-methyl methacrylate)/Iron Oxide Microparticles for Efficient Curcumin Delivery

**DOI:** 10.1021/acsomega.5c11646

**Published:** 2026-02-27

**Authors:** Ana B. da Silva, Suelen P. Facchi, Bruno R. Machado, Carlos F. Teodoro, Mazeyar P. Gashti, Ketul C. Popat, Adley F. Rubira, Elton G. Bonafé, Alessandro F. Martins

**Affiliations:** † Department of Chemistry, 42487 State University of Maringá (UEM), Maringá 87020-900, PR, Brazil; ‡ Laboratory of Materials, Macromolecules and Composites, Federal University of Technology - Paraná (UTFPR), Apucarana 86812-460, PR, Brazil; § Department of Bioengineering, College of Engineering and Computing, 3298 George Mason University, Fairfax, Virginia 22030, United States; ∥ Department of Chemistry, 6594 Pittsburg State University (PSU), Pittsburg, Kansas 66762, United States

## Abstract

This study successfully optimized the electrospraying process of poly­(butyl methacrylate-*co*-(2-dimethylamino)­ethyl methacrylate-*co*-methyl methacrylate) (P­(BMA-*co*-DMAEMA-*co*-MMA)) copolymer solutions containing curcumin (CUR) and iron oxide (Fe_3_O_4_) for the production of microparticles serving as carrier systems. P­(BMA-*co*-DMAEMA-*co*-MMA) is a cationic copolymer synthesized via free radical polymerization of the monomers N,N-dimethylaminoethyl methacrylate (DMAEMA), methyl methacrylate (MMA), and butyl methacrylate (BMA). By combining a pH-responsive poly­(methacrylate) matrix with superparamagnetic Fe_3_O_4_, this work addresses current limitations of CUR delivery systems (burst release and low drug loading). It provides a dual-stimuli platform for controlled release. P­(BMA-*co*-DMAEMA-*co*-MMA) solutions, with or without CUR and Fe_3_O_4_, were prepared in ethanol/N,N-dimethylformamide (EtOH/DMF) and processed via electrospraying. The copolymer concentration ranged from 10 to 30% (w/v) in EtOH/DMF (80/20 and 20/80 (v/v) ratios), and the presence of CUR (10, 20, and 30% (w/w)) and Fe_3_O_4_ (1–8% (w/w)). The influence of solution properties (viscosity, electrical conductivity, and surface tension) and processing conditions on particle morphology and drug loading was evaluated. The electrosprayed microparticles were analyzed using scanning electron microscopy (SEM), attenuated total reflectance Fourier-transform infrared spectroscopy (FTIR-ATR), X-ray diffraction (XRD), differential scanning calorimetry (DSC), and thermogravimetric analysis (TGA). Loading a significant amount of CUR in the microparticles was possible due to the solubility of CUR and copolymer in the EtOH/DMF mixture. The CUR crystallinity was significantly reduced compared to the loaded CUR. Controlled release studies of CUR were conducted and the sample prepared from a mixture containing 10% (w/v) of copolymer (COP), 20% (w/w) of CUR, and 8% (w/w) of Fe_3_O_4_ (sample COP10/CUR20/Fe_3_O_4_(8)), when exposed to an external magnetic field, significantly reduced the CUR release rate from 27.5%/h to 1.16%/h at pH 3.8 and 6.42%/h to 0.48%/h at pH 6.8. Release kinetics analysis indicated that the Korsmeyer–Peppas model best fitted the experimental data. These results demonstrate that Fe_3_O_4_-containing P­(BMA-*co*-DMAEMA-*co*-MMA) microparticles exhibit dual pH-responsive and magnetically responsive properties, resulting in a significant reduction of burst release and providing spatially and temporally tunable CUR delivery.

## Graphical abstract

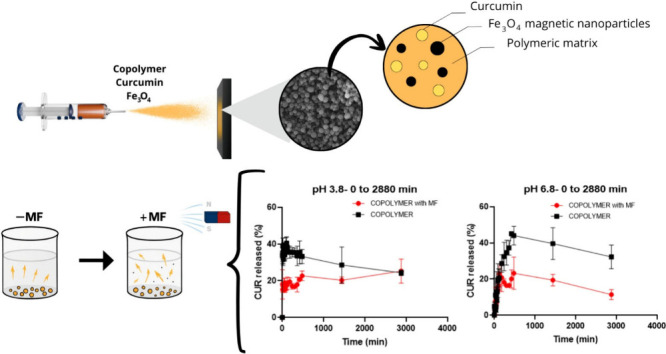

## 1 Introduction

Curcumin (CUR) ([(E,E)-1,7-bis­(4-hydroxy-3-methoxyphenyl)-1,6-heptadiene-3,5-dione]) is the main active compound in the perennial herb *Curcuma longa* (turmeric).^1^ It is a polyphenolic compound widely used as a food additive, known for its antioxidant, anti-inflammatory, and wound-healing properties.^2^ However, its poor absorption, rapid metabolism, and rapid elimination significantly compromise its *in vivo* stability, limiting its potential in pharmaceutical development.^3^ In addition, CUR exhibits high crystallinity, low water solubility (approximately 11 ng/mL), and poor stability in alkaline solutions and under light exposure. To overcome these limitations and improve its stability and bioavailability, several delivery systems have been developed, including bigels,^4^ inclusion complexes,^5^ hydrogels,^6^ composites,^7^ and liposomes.^8^ Despite these advances, many of these systems still exhibit limitations, such as low drug-loading capacity, initial burst release, instability in physiological media, and complex preparation routes.^9^ Thus, there remains a need for systems that combine high CUR loading, protection against degradation, and controlled release.

Using organic- or inorganic-based nano- and microparticles to encapsulate CUR can enhance its therapeutic efficacy. This strategy also prolongs its half-life, increases its solubility, and decreases its crystallinity.^10^ Among the main encapsulation techniques are precipitation,^11^ emulsification,^12^ coacervation,^13^ and electrospraying.^14^ In the specific case of CUR, nano- and microparticulate systems prepared by these techniques have shown a marked increase in apparent solubility and stability.^15^

This study employs electrospraying to develop CUR-loaded microparticles. This technique, known as electrohydrodynamic atomization, uses electrostatic forces to produce particles. The process involves injecting a polymeric solution or suspension containing the drug through a capillary needle connected to an electrically charged syringe. The solution or suspension is then directed toward a region of lower potential, represented by a grounded collector. During this trajectory, the solvent evaporates, and the formed particles are collected.^16^

This method stands out from conventional encapsulation approaches because it offers several advantages: it is a single-step process, requires smaller amounts of solvents or surfactants, eliminates the need for prolonged drying stages,^17^ and can be carried out at room temperature.^18^ Additionally, controlling parameters such as voltage, flow rate, solution concentration, and needle-to-collector distance allow for precise adjustment of particle diameter, morphology, and size distribution.^19^ This modulation promotes high encapsulation efficiencies, enabling the preparation of systems with high loadings of hydrophobic drugs, such as CUR.^20^ These features make electrospraying particularly suitable for the development of scalable controlled-release systems.

Controlling parameters such as concentration and voltage enables the production of particles capable of encapsulating the drug dispersed in the initial solution or suspension. For this purpose, polymers containing hydrophilic and hydrophobic groups are essential, as these features enable interaction with the solvent and hydrophobic drugs, such as CUR. This strategy can yield particles with the potential to function as controlled-release systems^21^ and protect drugs from factors that may compromise their integrity, such as light, heat, moisture, and pH variations.^22^ Moreover, when polyelectrolyte polymers bearing ionizable groups are used, pH-dependent responses can be exploited. In this process, the ionization of functional groups alters the matrix swelling and drug diffusion, thereby enabling modulation of the release profile across different physiological environments.^23^

In this context, the copolymer poly­(butyl methacrylate-*co*-(2-dimethylaminoethyl) methacrylate-*co*-methyl methacrylate) [P­(BMA-*co*-DMAEMA-*co*-MMA)] stands out as a biodegradable, cytocompatible, and cationic copolymer, owing to the presence of pendant secondary amine groups along its main chain.^24^ This copolymer is already employed in the pharmaceutical industry as a drug carrier matrix, with applications including odor and taste masking, as well as protection against light and moisture.^25,26^ Due to these promising properties, P­(BMA-*co*-DMAEMA-*co*-MMA) was selected for particle production via electrospraying in the present study, aiming to develop controlled-release systems for CUR. The combination of the copolymer’s cationic, pH-sensitive nature with the versatility of electrospraying provides an attractive platform to tune morphology, drug loading, and pH-responsive behavior within a single drug delivery system.

P­(BMA-*co*-DMAEMA-*co*-MMA) particles are pH-responsive; however, studies indicate that this stimulus alone is insufficient to prevent rapid drug release and achieve a sustained release profile. To address this limitation, composite particles of P­(BMA-*co*-DMAEMA-*co*-MMA) incorporating iron oxide (Fe_3_O_4_) were developed, imparting magnetic properties to the system and enabling responsiveness to an external magnetic field. When exposed to magnetic fields, magnetic composite materials often exhibit a slower release profile, which is attributed to the increased tortuosity of the drug diffusion pathway.^27^ Moreover, Fe_3_O_4_ stands out for its biocompatibility, superparamagnetic behavior, and high surface area.^28,29^ Compared to other nonmagnetic metal oxides, Fe_3_O_4_ offers the crucial advantage of enabling modulation of drug release through external magnetic fields, thereby allowing, in principle, spatially and temporally controlled release at specific targets.^29,30^ It is a material that has been extensively studied for biomedical applications, with low cytotoxicity and a well-established history of use in contrast agents and drug delivery systems, making it particularly attractive as a dispersed phase in polymeric composites for CUR administration.^31^

This study presents the optimization of the electrospraying process for P­(BMA-*co*-DMAEMA-*co*-MMA)/CUR mixtures, with or without Fe_3_O_4_, in a mixture of EtOH/DMF, to obtain composite particles with both magnetic and pH-responsive properties. The particles were *in situ* encapsulated with up to 30% (w/w) CUR and combined with up to 8% (w/w) Fe_3_O_4_. The materials were characterized by Scanning Electron Microscopy (SEM), Fourier Transform Infrared Spectroscopy (FTIR), Differential Scanning Calorimetry (DSC), Thermogravimetric Analysis (TGA), and X-ray Diffraction (XRD). CUR release assays were performed under different pH conditions, both in the presence and absence of an external magnetic field. The CUR release mechanism was investigated by applying nonlinear kinetic models to the experimental release profiles. To our knowledge, this is the first time that a study on optimizing and producing P­(BMA-*co*-DMAEMA-*co*-MMA)/Fe_3_O_4_ composite particles for an efficient CUR delivery system is presented.

## 2 Materials and Methods

The poly­(butyl methacrylate-*co*-(2-dimethylaminoethyl) methacrylate-*co*-methyl methacrylate) copolymer [P­(BMA-*co*-DMAEMA-*co*-MMA)] (47,000 g/mol) was provided by Evonik (Barcelona, Spain). Curcumin (CUR) from *Curcuma longa* (Turmeric), iron oxide (Fe_3_O_4_) (97%) (50 nm), Pluronic F-127 (12,600 g/mol), and N,N-dimethylformamide (DMF) (99.8%) were obtained from Sigma-Aldrich (São Paulo, Brazil). Ethyl alcohol (EtOH) (99.8%) was acquired from Synth (São Paulo, Brazil).

### 2.1 Preliminary Electrospraying Tests

The methodology used to obtain the composite particles was based on a previously described procedure, with some modifications.^32^ Scheme 1 illustrates the *in situ* process for loading electrosprayed particles with CUR and Fe_3_O_4_. Solutions of P­(BMA-*co*-DMAEMA-*co*-MMA) in EtOH/DMF, at volumetric ratios of 80/20 and 20/80 (v/v), were prepared at different copolymer concentrations (10, 12, 14, 18, 22, 26, and 30% (w/v)) (Table 1). The sample designated “COP10­(DMF80)” refers to particles produced from a 10% (w/v) copolymer solution prepared by electrospraying a solution containing 80% DMF by volume. The notation “COP10­(EtOH80)” indicates particles obtained from a 10% (w/v) copolymer solution prepared by electrospraying a solution containing 80% EtOH by volume. The same naming convention was applied to the other samples (Table 1).

**Table 1:** Experimental Conditions Used to Prepare P­(BMA-*co*-DMAEMA-*co*-MMA) Particles via Electrospraying of 5.0 mL of Solution

Sample	P(BMA-*co*-DMAEMA-*co*-MMA) (g)	Concentration % (w/v)	EtOH/DMF % (v/v)
COP10(DMF80)	0.50	10	20/80
COP12(DMF80)	0.60	12	20/80
COP14(DMF80)	0.70	14	20/80
COP18(DMF80)	0.90	18	20/80
COP22(DMF80)	1.10	22	20/80
COP26(DMF80)	1.30	26	20/80
COP30(DMF80)	1.50	30	20/80
COP10(EtOH80)	0.50	10	80/20
COP12(EtOH80)	0.60	12	80/20
COP14(EtOH80)	0.70	14	80/20
COP18(EtOH80)	0.90	18	80/20
COP22(EtOH80)	1.10	22	80/20
COP26(EtOH80)	1.30	26	80/20
COP30(EtOH80)	1.50	30	80/20

**Scheme 1:**
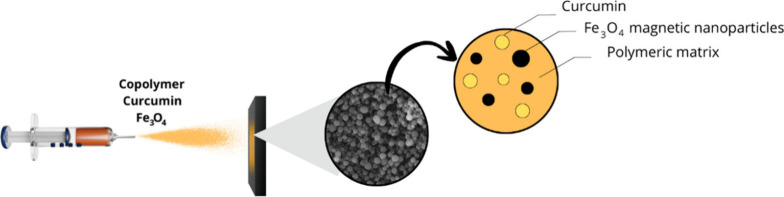
Representation of the Electrospraying Process of the Copolymer + Curcumin + Fe_3_O_4_ Suspension

The operational parameters for the electrospraying process were a voltage of 12 kV and a flow rate of 0.5 mL/h, controlled using an infusion pump (Harvard 2.2.2, USA). The static metallic collector consisted of a copper plate covered with aluminum foil. The 10 mL syringe was connected to a capillary needle (14G; 2.1 × 40 mm), with a fixed distance of 10 cm between the needle tip and the collector. All experiments were performed at room temperature (25 °C).

### 2.2 Preparation of Composite Particles by Electrospraying

Preliminary results indicated that the experimental condition using 10% (w/v) copolymer in an EtOH/DMF ratio of 80/20 (v/v) (COP10­(EtOH80)) produced the most homogeneous particles with the smallest average diameter. For this reason, this condition was selected to prepare composite particles incorporating CUR (Table 2). The preliminary test evaluated CUR concentrations of 10%, 20%, and 30% (relative to the copolymer mass).

**Table 2:** Experimental Conditions Used for the Preparation of Composite Particles via Electrospraying of 5.0 mL of P­(BMA-*co*-DMAEMA-*co*-MMA)/CUR and P­(BMA-*co*-DMAEMA-*co*-MMA)/CUR/Fe_3_O_4_/Pluronic F-127 Mixtures

Sample	P(BMA-*co*-DMAEMA-*co*-MMA) % (w/v)	EtOH/DMF % (v/v)	CUR % (w/w)	Fe_3_O_4_ % (w/w)	F-127 % (w/w)
COP/CUR20/Fe_3_O_4_(1)	10	80/20	20	1	0.10
COP/CUR20/Fe_3_O_4_(2)	10	80/20	20	2	0.10
COP/CUR20/Fe_3_O_4_(4)	10	80/20	20	4	0.10
COP/CUR20/Fe_3_O_4_(8)	10	80/20	20	8	0.10

After solubilizing the copolymer and CUR, iron oxide (Fe_3_O_4_) and Pluronic F-127 (0.1% (w/w)) were added to the mixture. Pluronic F-127 was used to promote the dispersion of Fe_3_O_4_ nanoparticles within the P­(BMA-*co*-DMAEMA-*co*-MMA)/CUR solution. The mixture was subjected to an ultrasonic bath for 3 min and then electrosprayed under the same experimental conditions described in Section 2.1.

The experimental condition “COP/CUR20/Fe_3_O_4_(1)” represents a composition of 10% (w/w) P­(BMA-*co*-DMAEMA-*co*-MMA), 20% (w/w) CUR, and 1% (w/w) Fe_3_O_4_. This nomenclature also applies to the other sample codes. All conditions listed in Table 2 include 0.1% (w/w) Pluronic F-127 in their composition. The iron oxide concentration was varied between 1% and 8%; higher concentrations resulted in needle clogging, negatively impacting the electrospraying process. All experiments were performed at room temperature (25 °C).

### 2.3 Characterization

The conductivity, viscosity, and surface tension of the P­(BMA-*co*-DMAEMA-*co*-MMA) solutions were measured at 25 °C. Conductivity was determined using an MS Tecnpon conductimeter with a cell constant (k) of 1. Viscosity was measured from flow time using a Ubbelohde viscometer, and surface tension was evaluated with a Lecomte Du Noüy K6 apparatus at 25 °C.

Particle characterization was performed using scanning electron microscopy (SEM) on a Shimadzu SS 550 instrument operated at 12.5 kV. Before analysis, the samples were coated with a thin layer of gold (10 nm). Fourier-transform infrared spectroscopy (FTIR-ATR) was performed using a Shimadzu 8300 spectrometer (Japan), covering the range of 4000–500 cm^–1^ with 64 scans. Thermogravimetric analyses (TGA/DTG) were performed on a Shimadzu TGA50 analyzer at a heating rate of 10 °C/min from 25 to 650 °C under an argon atmosphere. Differential scanning calorimetry (DSC) was conducted with a Shimadzu DSC60 Plus calorimeter at a heating rate of 10 °C/min from 200 to 300 °C, under an argon purge (50 mL/min).

For the study of X-ray diffraction (XRD) patterns, a D2 Phaser diffractometer (Bruker) equipped with a Cu–Kα_1_ X-ray source (1.54 Å) and a power of 300 W (30 kV × 10 mA) was used. Patterns were collected over a 2θ range of 3° to 60°, with a step size of 0.033° and a scanning rate of 2°/min.

### 2.4 *In Vitro* CUR Release Assays

The electrosprayed particles were easily removed from the collector and weighed. It was assumed that all CUR added to the copolymer and copolymer/Fe_3_O_4_ mixtures was efficiently encapsulated within the particles. Cumulative curcumin (CUR) release assays were then conducted following a previously described methodology, with adaptations.^33^ CUR release was evaluated in simulated intestinal fluid (SIF, pH 6.8) and sodium acetate/acetic acid buffer (pH 3.8). Particles (6.2 mg) encapsulating CUR were placed in sealed flasks containing 150 mL of SIF or buffer solution. The flasks were maintained under orbital shaking (100 rpm) at 37 °C for 48 h.

At specific time intervals, 1.5 mL aliquots were withdrawn from the solutions and immediately diluted with 1.5 mL of ethanol for subsequent analysis by UV–vis spectrophotometry at 425 nm. A CUR calibration curve was prepared in a water/ethanol mixture (1:1) over a concentration range of 1–10 mg/L and used to quantify the amount of CUR released from the particles. The assays were performed in triplicate, both with and without the application of an external magnetic field. For this purpose, magnets were attached to the outside of the flasks containing SIF or the sodium acetate/acetic acid buffer solution, allowing evaluation of the magnetic field’s influence on CUR release from the encapsulated system. A control assay was performed with pure CUR (nonloaded), adding the drug to buffers (8.26 mg/L) and measuring the free CUR absorbance over 48 h.

### 2.5 CUR Release Mechanism

The CUR release mechanism was analyzed by applying different kinetic models to the experimental release curves. The models employed were Zero-Order, Pseudo-First-Order, Korsmeyer-Peppas, and Higuchi. These models were fitted to the experimental data to determine the kinetic parameters and evaluate the CUR release mechanism. The coefficients of determination (*R* ^2^) obtained for each model were compared to assess their relevance in the context of the studied system from these fittings. This approach provides insights into the physical and chemical processes responsible for CUR release under the investigated experimental conditions.

### 2.6 Statistical Analysis

Statistical analysis was performed using ANOVA followed by Tukey’s test, with a significance level set at 5% (GraphPad Prism 6.0, GraphPad Software).

## 3 Results and Discussion

### 3.1 Conductivity, Viscosity, and Surface Tension

Conductivity, viscosity, and surface tension were evaluated for P­(BMA-*co*-DMAEMA-*co*-MMA) solutions at concentrations of 10, 18, and 26% (w/v) in binary solvent mixtures of EtOH/DMF at ratios of 80/20 and 20/80 (v/v) (Table 3). These measurements are primarily influenced by the EtOH/DMF ratio, the type of polymer, the solvent used, and the concentration.^32^

**Table 3:** Conductivity, Viscosity, and Surface Tension Values of the Solvents EtOH and DMF, as Well as of P­(BMA-*co*-DMAEMA-*co*-MMA) Solutions Prepared in Binary EtOH/DMF Mixtures at 80/20 and 20/80 (v/v) Ratios^a^

Sample	Conductivity (μS/cm)	Viscosity (N·s/m^2^)	Surface Tension (mN/m)
EtOH	1.4 × 10^–9^ ^a^	0.00108^a^	0.0223^a^
DMF	6.0 × 10^–9^ ^a^	0.00082^a^	0.0035^a^
COP10(DMF80)	5.72 ± 0.02 a	0.00340	41.23 ± 1.50
COP18(DMF80)	5.38 ± 0.18 a	0.00810	40.1 ± 0.76
COP26(DMF80)	5.57 ± 0.06 a	0.01960	40.5 ± 0.55
COP10(EtOH80)	5.20 ± 0.07 ab	0.00400	35.0 ± 1.76
COP18(EtOH80)	5.29 ± 0.07 ab	0.01200	36.5 ± 1.32
COP26(EtOH80)	4.88 ± 0.07 b	0.03000	39.9 ± 0.95

a Different lowercase letters in the “Conductivity” column indicate results with significant differences (*p* ≤ 0.05).

b Conductivity, viscosity, and surface tension measurements according to Smallwood.^34^

The polymer solutions exhibited a slight variation in conductivity (ranging from 4.88 ± 0.07 to 5.57 ± 0.06 μS/cm), indicating that this parameter likely has a minimal influence on the electrospraying process. In contrast, solutions with a higher concentration of EtOH showed higher viscosity and lower surface tension, suggesting that EtOH is a more suitable solvent for the copolymer P­(BMA-*co*-DMAEMA-*co*-MMA).

According to Abdulhussain et al.,^35^ an ideal solvent should efficiently dissolve the polymer, exhibit moderate volatility, and ensure that the solution has appropriate viscosity and surface tension to allow polymer jet formation and stretching in the production of nanofibers, or droplet formation in particle electrospraying.

The comparison between the experimental conditions COP10­(EtOH80) and COP10­(DMF80) shows that the solution with a higher volume percentage of EtOH (80% volume) exhibits higher viscosity (0.004 N·s/m^2^) and lower surface tension (35 mN/m). Similarly, when comparing the mixtures COP18­(EtOH80) and COP18­(DMF80), an increased fraction of EtOH leads to higher viscosity and lower surface tension.

Furthermore, increasing the copolymer concentration also increases solution viscosity. For example, comparing the conditions COP10­(EtOH80) and COP26­(EtOH80), viscosity increased from 0.004 N·s/m^2^ to 0.030 N·s/m^2^. The same trend is observed between COP10­(DMF80) and COP26­(DMF80), where viscosities increased from 0.0034 N·s/m^2^ to 0.0196 N·s/m^2^, respectively. This increase is attributed to a higher concentration of polymer chains in solution, which promotes chain entanglement and increases viscosity.^36^

### 3.2 Morphology of Electrosprayed Particles: Preliminary Results

Figure 1 shows SEM images of materials obtained by electrospraying P­(BMA-*co*-DMAEMA-*co*-MMA) solutions at concentrations of 10, 12, 14, 18, 22, 26, and 30% (w/v), using an EtOH/DMF solvent mixture at a 20/80 (v/v) ratio. No fiber formation was observed for concentrations of 10, 12, 14, 18, and 22% (w/v), indicating that electrospraying prevailed over electrospinning.^37^ Under these conditions, only particles, specifically microparticles, were formed, with average diameters ranging from 704  ± 246 to 1191  ±  589 nm (Figure 1).

**Figure 1:**
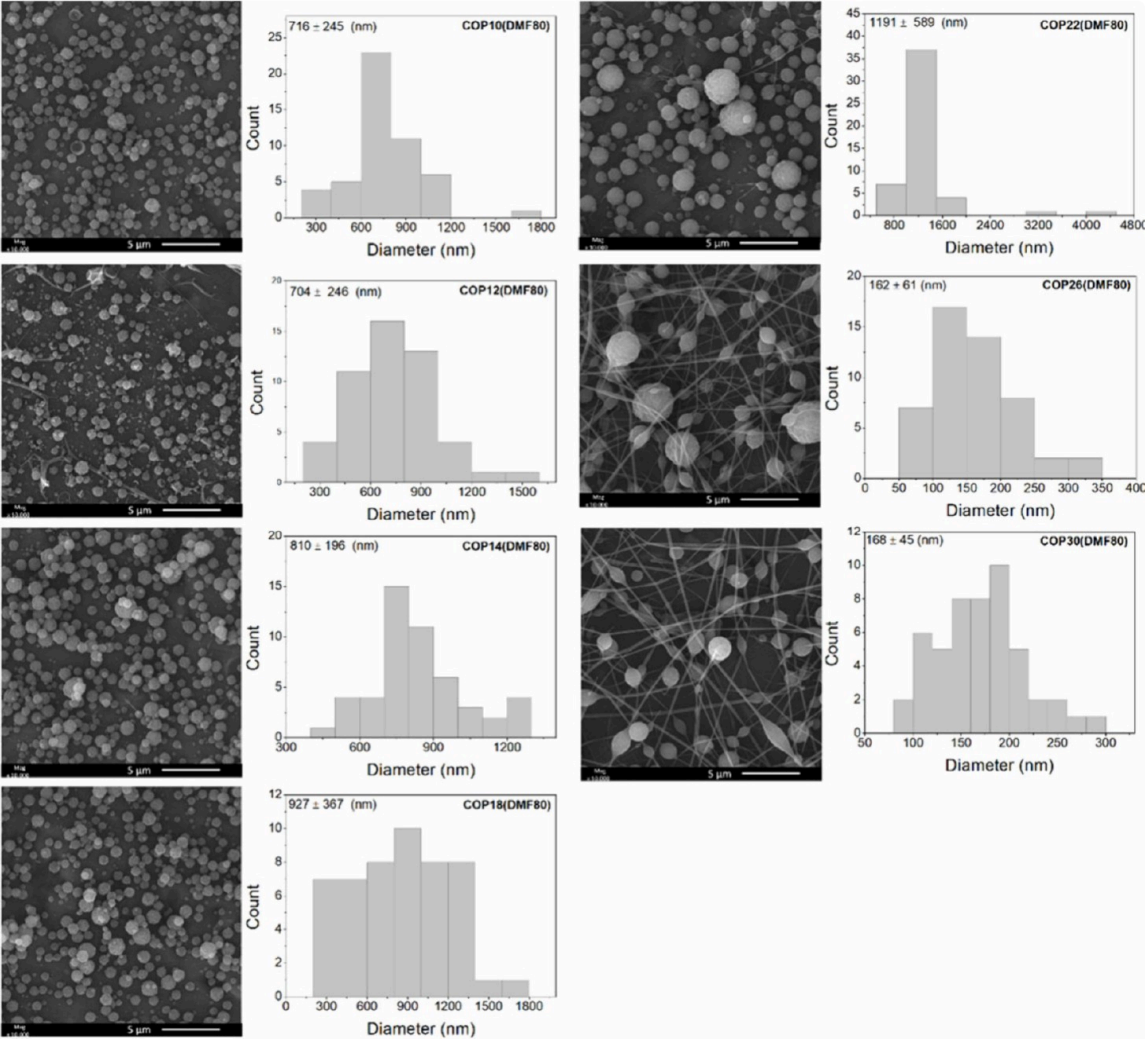
SEM images of P­(BMA-*co*-DMAEMA-*co*-MMA)-based materials produced from solutions with a higher proportion of DMF (80% (v/v)), obtained by electrospraying the solutions listed in Table 1.

The experimental conditions with 10, 12, 14, and 18% (w/v) of P­(BMA-*co*-DMAEMA-*co*-MMA) in 80% volume of DMF showed no significant differences in the mean particle diameter (*p* ≤ 0.05), suggesting that slight variations in copolymer concentration do not substantially affect particle diameter. The absence of fibers in these samples is due to the low viscosity of the polymer solution, which is insufficient to sustain continuous-jet formation during electrospinning.

According to Haider et al.,^36^ when the solution has low viscosity, the applied electric field, combined with surface tension, can break the solution jet into droplets before it reaches the collector, resulting in particles or beaded fibers. Conversely, at concentrations of 26% and 30% (w/v), the increased viscosity favored electrospinning, resulting in continuous fibers without beads. In these cases, fibers with average diameters of 162  ±  61 nm and 168  ±  45 nm were obtained, respectively. In this study, the focus is not on fibers but on the microparticles produced by electrospraying.

Figure 2 shows SEM images of samples obtained from solutions prepared with an EtOH/DMF solvent ratio of 80/20 (v/v) and copolymer concentrations of 10, 12, 14, 18, 22, 26, and 30% (w/v). Under higher EtOH volume percentages and copolymer concentrations of 10, 12, 14, and 18% (w/v), microparticles were obtained with average diameters ranging from 474 ±  235 nm to 969  ±  340 nm, showing statistically significant differences (*p* ≤ 0.05). As the copolymer concentration increased, the process transitioned from electrospraying to electrospinning. Fiber formation began at a copolymer concentration of 22%, although uniform fibers were only yielded without beads at 30%. At 22% and 26% (w/v) concentrations, fibers exhibited beads along their length, with average diameters of 155  ±  97 nm and 244  ± 70 nm, respectively (Figure 2).

**Figure 2:**
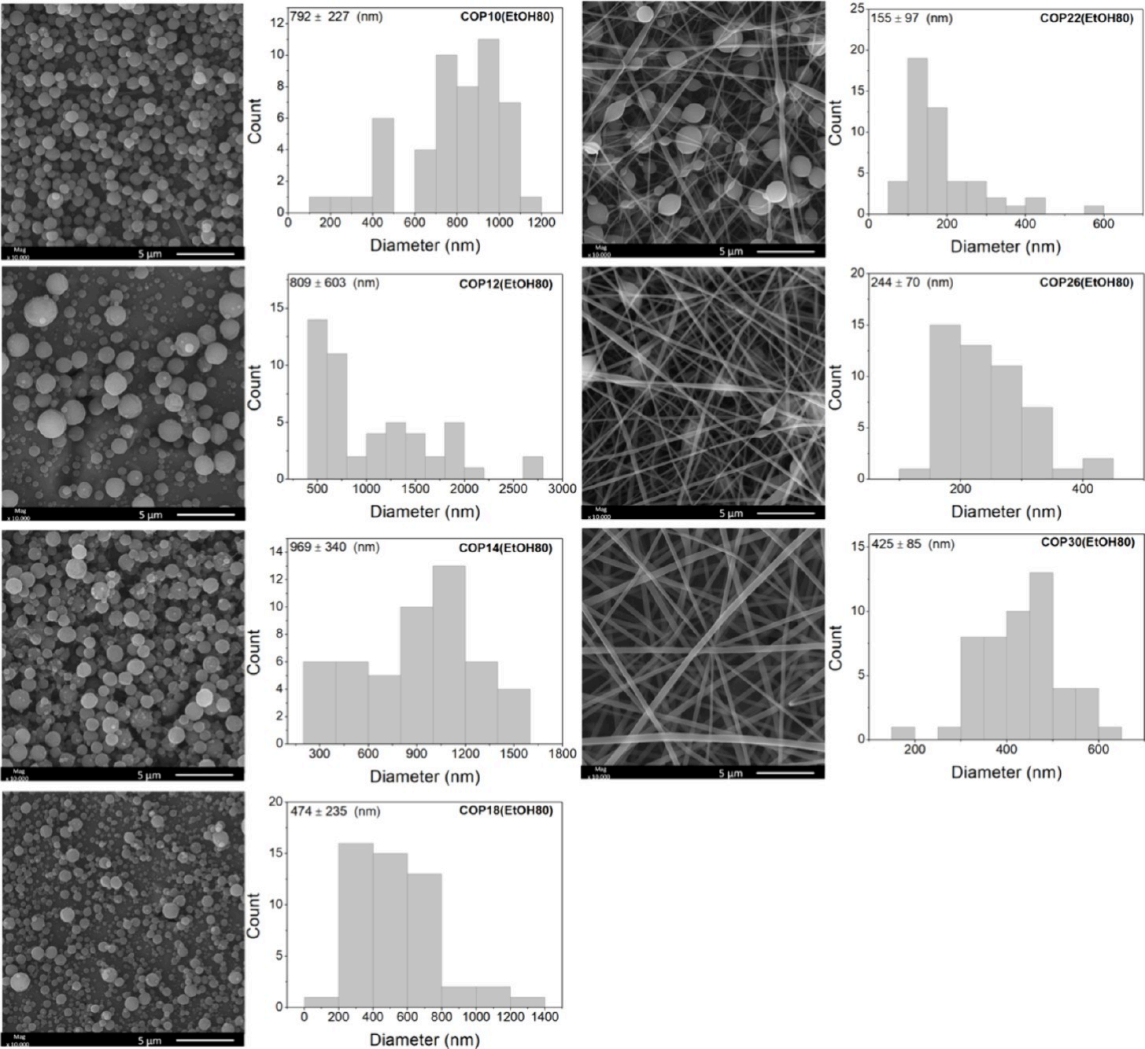
SEM images of P­(BMA-*co*-DMAEMA-*co*-MMA)-based materials obtained from copolymer solutions with higher EtOH content (80% volume) after electrospinning and/or electrospraying under the experimental conditions listed in Table 1.

The experimental condition with 30% copolymer yielded fibers with. An appropriate morphology and no beads, exhibiting an average diameter of 425  ± 85 nm. As previously mentioned, higher copolymer concentrations increase solution viscosity. Consequently, polymer solutions with higher viscosity are less likely to form beads along the fibers or display inadequate morphology.^38^ With increasing viscosity, the polymer droplets tend to elongate, forming smooth fibers.^39^

Comparing the experimental conditions COP30­(EtOH80) and COP30­(DMF80), it is observed that fibers obtained from solutions with a higher EtOH content exhibit more homogeneous morphology. In contrast, at the same copolymer concentration, fibers produced from solutions with higher DMF content showed beads. These results indicate that EtOH is a more suitable solvent for the copolymer than DMF. To avoid structural defects, such as bead formation, it is essential to ensure compatibility between the polymer and the solvent.^35^ In this study, electrospraying occurred at copolymer concentrations of 15–20% (w/v). As the copolymer concentration increased, a transition from electrospraying to electrospinning was observed, with fiber formation starting only at 25% P­(BMA-*co*-DMAEMA-*co*-MMA).^40^

### 3.3 Morphology of CUR-Loaded Particles

Based on the preliminary tests, the experimental condition COP10­(EtOH80) was selected to prepare electrosprayed particles loaded with CUR. This condition was chosen because it allowed the formation of homogeneous particle spheres using a lower copolymer concentration. The encapsulation test was conducted by varying the CUR concentration at 10%, 20%, and 30% (w/w) relative to the copolymer mass in the solution. SEM images of the resulting particles are shown in Figure 3.

**Figure 3:**
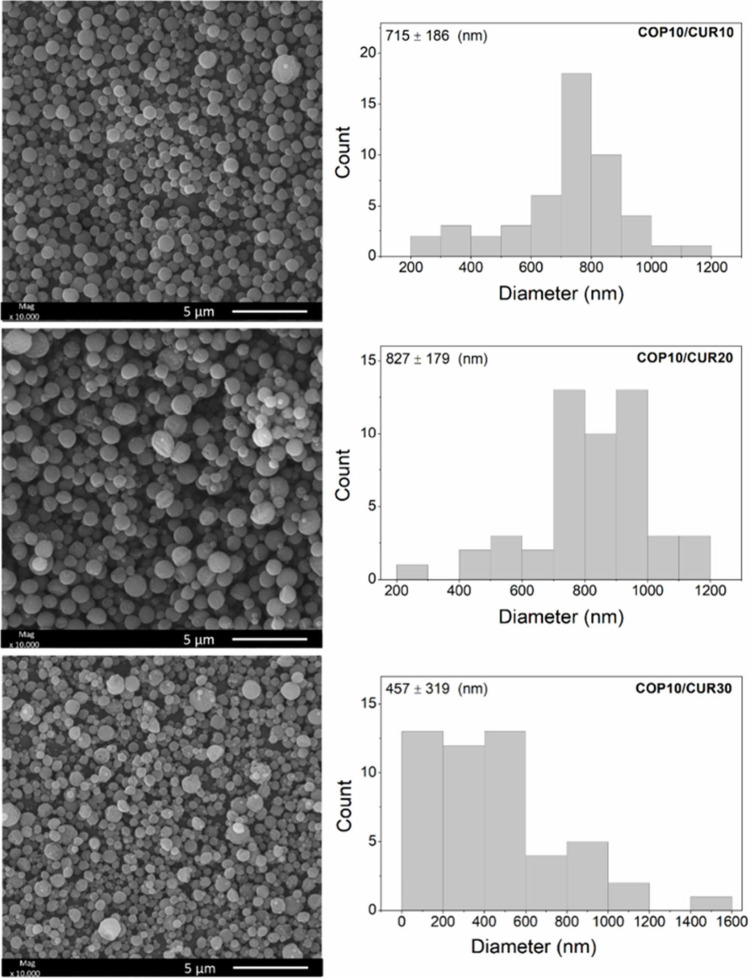
SEM images of the particles obtained under the optimized experimental condition COP10­(EtOH80) are shown. In these samples, CUR was incorporated into the solutions at concentrations of 10, 20, and 30% (w/w) relative to the copolymer mass, followed by electrospraying. The COP10/CUR10 sample was prepared from a solution containing 10% (w/v) copolymer, 10% CUR (w/w), and EtOH/DMF as solvent in an 80/20 (v/v) ratio. Similarly, the COP10/CUR20 and COP10/CUR30 samples were prepared with 10% (w/v) copolymer, 20% and 30% CUR (w/w), respectively, using the same solvent ratio.

The experimental conditions for CUR incorporation (10%, 20%, and 30% (w/w)) led to the formation of spherical particles with mean diameters ranging from 457 ± 319 nm to 827 ± 179 nm, showing a statistically significant difference (*p* ≤ 0.05). The COP10/CUR30 sample, which contained the highest CUR concentration, exhibited a mean diameter of 457 ± 319 nm and lower particle size uniformity than the COP10/CUR20 sample, which had a mean diameter of 827 ± 179 nm.

These findings are in accordance with other results. For example, Gómez-Estaca et al.^41^ developed an electrosprayed material for CUR encapsulation to enhance its water solubility and stability. The polymeric solution was prepared using gelatin and solvents of ethanol, distilled water, and acetic acid, containing 10% (w/w) CUR. The system achieved a 100% encapsulation efficiency. After encapsulation, CUR solubility increased by 38.6-fold. The antioxidant properties of the encapsulated CUR were significantly higher (*p* ≤ 0.05) than those of free CUR, as demonstrated by ferric reducing capacity and ABTS radical scavenging tests. Nonencapsulated CUR showed negligible antibacterial activity at concentrations up to 100 mg/mL, whereas gelatin-encapsulated CUR at 4 mg/mL reduced microbial populations by 2.08, 1.67, 2.70, and 2.18 log CFU/mL against *L. monocytogenes*, *S. enterica*, *S. aureus*, and *E. coli*, respectively.

In the study by Pires et al. CUR was encapsulated at concentrations of 0.50, 0.75, and 1% (w/w relative to starch) using electrospinning and electrospraying techniques. Polymeric solutions were prepared with starch concentrations of 3%, 5%, 10%, 15%, 20%, and 25% (w/v) in 75% formic acid (v/v in ultrapure water), and the solutions were stirred. The solutions were then allowed to rest at 25 ± 2 °C for 24 or 48 h without agitation before electrospinning or electrospraying. The optimal electrospraying condition was achieved with 10% starch and 48 h of resting, yielding particles with mean diameters ranging from 1373 to 1787 nm. CUR encapsulation efficiency ranged from 79.01% to 97.09%. Encapsulated CUR exhibited greater thermal stability at 180 °C for 2 h than nonencapsulated CUR. The lowest percentage loss for encapsulated CUR was 13.05% (in the 1% CUR sample), whereas nonencapsulated CUR showed a loss of 53.69% after the same thermal treatment.^42^

### 3.4 Morphology of Curcumin-Loaded Composite Particles

To incorporate Fe_3_O_4_ into the particles for the development of systems responsive to both pH and the application of an external magnetic field, the experimental condition corresponding to the COP10/CUR20 sample was selected due to its higher homogeneity, considering the amount of CUR incorporated. Figure 4 presents SEM images of electrosprayed particles obtained from copolymer solutions containing 20% (w/w) CUR and varying Fe_3_O_4_ concentrations (1%, 2%, 4%, and 8% (w/w)).

**Figure 4:**
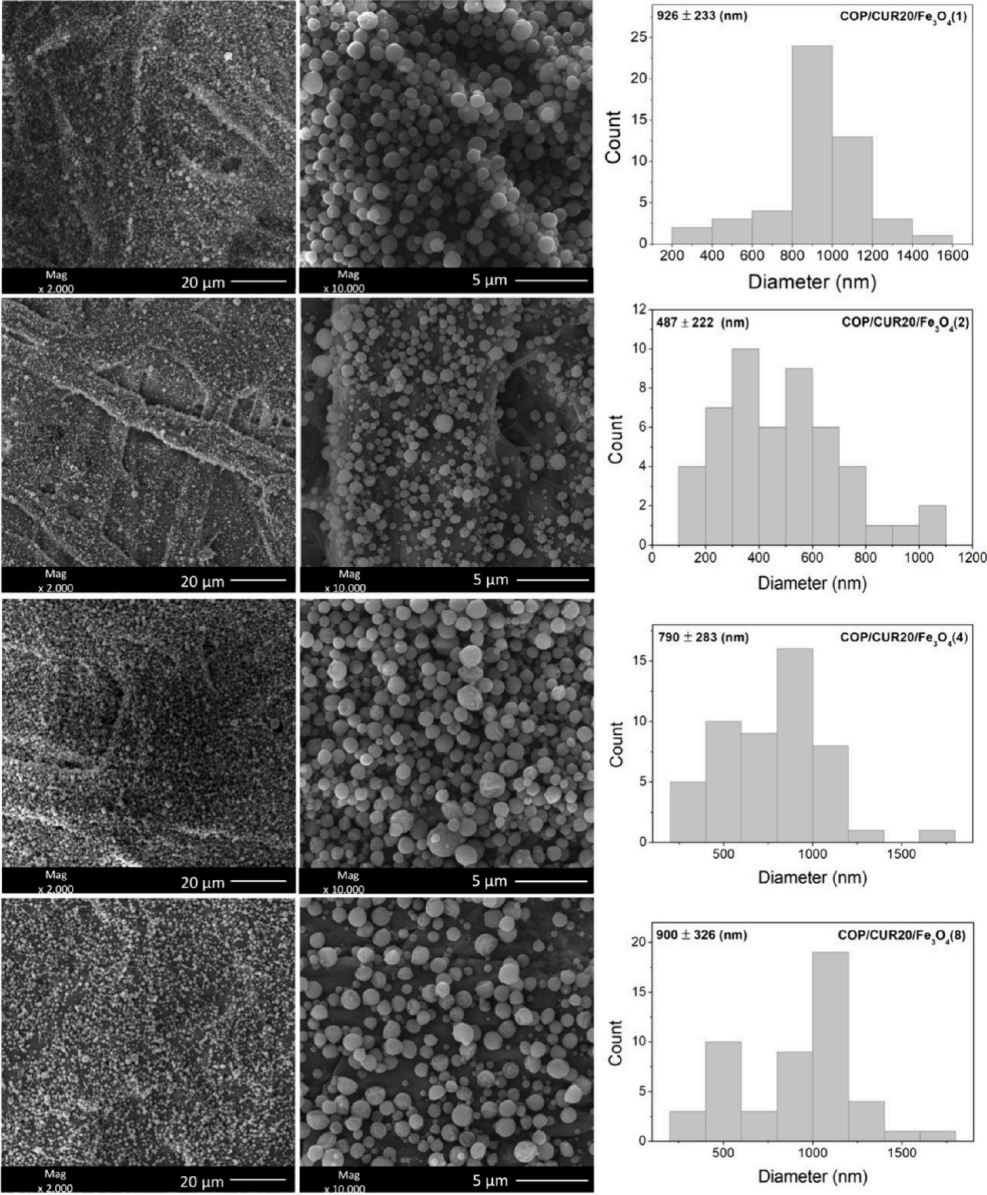
SEM images of electrosprayed COP10/CUR20 microparticles (10% copolymer and 20% CUR) loaded with Fe_3_O_4_ at concentrations of 1%, 2%, 4%, and 8% (w/w relative to the copolymer mass). The sample COP/CUR20/Fe_3_O_4_(1) was obtained from a solution containing 10% (w/v) copolymer, 20% CUR (w/w), 1% Fe_3_O_4_ (w/w), and an EtOH/DMF solvent mixture (80/20 (v/v)). This nomenclature applies similarly to samples COP/CUR20/Fe_3_O_4_(2), COP/CUR20/Fe_3_O_4_(4), and COP/CUR20/Fe_3_O_4_(8).

The incorporation of Fe_3_O_4_ at concentrations of 1%, 2%, 4%, and 8% did not prevent the formation of CUR-loaded particles. However, the presence of Fe_3_O_4_ not only promoted microparticle formation and enhanced their organization into fibrous structures (Figure 4). This organization was most evident at 2% Fe_3_O_4_, with particles exhibiting an average diameter of 487 ± 222 nm, a statistically significant difference compared to the diameters observed at 1%, 4%, and 8% Fe_3_O_4_ (*p* ≤ 0.0001). In contrast, in the sample with 8% Fe_3_O_4_, this organization was no longer apparent, and the average particle diameter increased to 900 ± 326 nm (Figure 4).

Li et al.^43^ developed multifunctional chitosan microspheres, produced via electrospraying, incorporating Fe_3_O_4_ and graphene oxide for controlled drug release. Doxorubicin was used as a model drug and incorporated into the matrix by either direct addition to the solution or postadsorption. The resulting microspheres had average diameters ranging from 100 to 1100 μm. The presence of Fe_3_O_4_ imparted magnetic properties, enabling remote drug release via near-infrared (NIR) light and ultrasonic irradiation. Ultrasonication increased the drug release rate by approximately 10%, an effect attributed to thermally induced vibrations in the microspheres, facilitating the diffusion of the encapsulated drug molecules.

Rasekh et al.^44^ employed a coaxial electrospraying method to encapsulate genistein (a model drug), Fe_3_O_4_ nanoparticles, and a fluorophore (fluorescent dye) within a layered particulate system using a triestearin-based lipid shell. The aim was to develop a material with potential for simultaneous disease diagnosis and treatment. Coaxial electrospraying enabled the formation of particles with diameters ranging from 0.65 to 1.2 μm, efficiently encapsulating Fe_3_O_4_ nanoparticles and achieving approximately 92% encapsulation efficiency for genistein. The system exhibited a triphasic drug release profile. Moreover, the incorporation of Fe_3_O_4_ resulted in a significantly slower drug release over 30 h.

### 3.5 Characterization

The FTIR-ATR spectra of CUR and P­(BMA-*co*-DMAEMA-*co*-MMA) are shown in Figure 5A, while Figure 5B presents the spectra of the microparticles COP10­(EtOH80), COP10/CUR20, COP/CUR20/Fe_3_O_4_(1), and COP/CUR20/Fe_3_O_4_(8).

**Figure 5:**
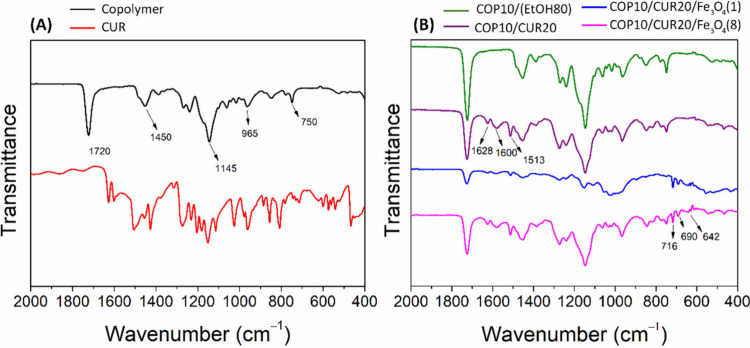
FTIR-ATR spectra. (A) Copolymer P­(BMA-*co*-DMAEMA-*co*-MMA), CUR, and electrosprayed microparticles COP10­(EtOH80) and COP10/CUR20. (B) Composite microparticles COP/CUR20/Fe_3_O_4_(1) and COP/CUR20/Fe_3_O_4_(8).

The spectra of P­(BMA-*co*-DMAEMA-*co*-MMA) and the COP10­(EtOH80) sample exhibit similar spectral features. Prominent bands include the C=O axial stretching of ester groups at 1720 cm^–1^,^45^ the axial deformation of the C–N bond of tertiary aliphatic amines at 1145 cm^–1^, and the angular deformations of C–H in the copolymer chains at 1450, 965, and 750 cm^–1^.^46^

The FTIR spectra of the COP10/CUR20 microparticles exhibit characteristic bands similar to those observed in the copolymer and the COP10­(EtOH80) sample. In addition, bands confirming the presence of CUR in the composition are highlighted, such as the band at 1628 cm^–1^, attributed to the symmetric C=C stretching vibrations in aromatic rings,^47^ and the band at 1513 cm^–1^, corresponding to the axial deformation vibrations of the C–C bonds in the CUR rings.^48^

The FTIR spectra of the composite microparticles COP/CUR20/Fe_3_O_4_(1) and COP/CUR20/Fe_3_O_4_(8) display the same characteristic bands observed in the COP10/CUR20 sample. However, they exhibit a band at 642 cm^–1^, attributed to Fe–O stretching.^49^ In addition, the microparticles containing Fe_3_O_4_ show new vibration bands in the low-frequency region (at 690 and 716 cm^–1^), which can be assigned to the Fe–O bonds of the magnetite particles.^50^

### 3.6 Thermal Analyses

DSC curves for CUR, P­(BMA-*co*-DMAEMA-*co*-MMA) copolymer, and the microparticles COP10­(EtOH80), COP10/CUR20, and COP/CUR20/Fe_3_O_4_(8) are presented in Figure 6. The curves display endothermic peaks associated with water evaporation between 52 and 66 °C. The copolymer and COP10­(EtOH80) DSC curve profiles show two endothermic peaks at 302 and 406 °C, whereas the COP10/CUR20 profile exhibits slightly shifted peaks with reduced intensity. The CUR DSC curve shows a sharp endothermic peak at 178 °C, attributed to the CUR crystalline structure.^51^

**Figure 6:**
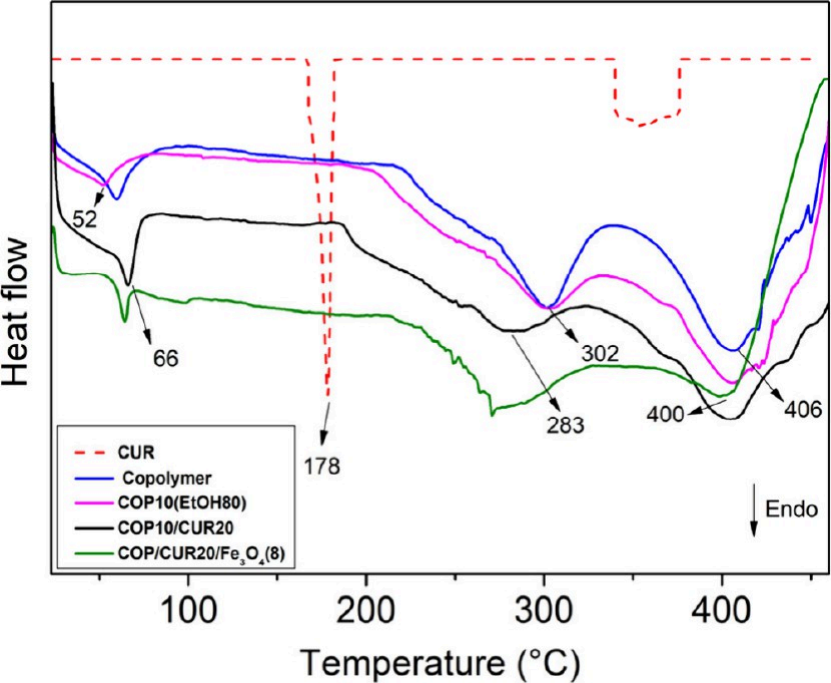
DSC curves of the P­(BMA-*co*-DMAEMA-*co*-MMA) copolymer, CUR, and microparticles COP10­(EtOH80), COP10/CUR20, and COP10/CUR20/Fe_3_O_4_(8).

The COP10/CUR20 did not exhibit an endothermic peak near the melting point of CUR, indicating that interaction with the copolymer matrix altered the CUR crystallinity into an amorphous state.^1^ The reduction in the melting peak intensity suggests that the copolymer matrix decreased the crystallinity of the CUR.^52^ Notably, the concentration of CUR in the microparticles is 20% (w/w), which is considerably higher than in previous studies that demonstrated a reduction in CUR crystallinity within polymeric matrixes.^33,52^ The DSC profile of the Fe_3_O_4_-containing sample shows endothermic peaks similar to those of COP10/CUR20, with slight exothermic peak shifts between 407 and 457 °C.

The TGA/DTG curves of the P­(BMA-*co*-DMAEMA-*co*-MMA) copolymer and COP10­(EtOH80) microparticles show weight alteration starting at 273 °C, indicating that no significant degradation occurs below this temperature (Figure 7).^53^ The curves display two main weight-change events: the first, occurring between 273 and 354 °C, is associated with the removal of dimethylamino groups and the formation of six-membered cyclic anhydrides; the second, starting at 393 °C, corresponds to the complete degradation of the copolymer.^54^

**Figure 7:**
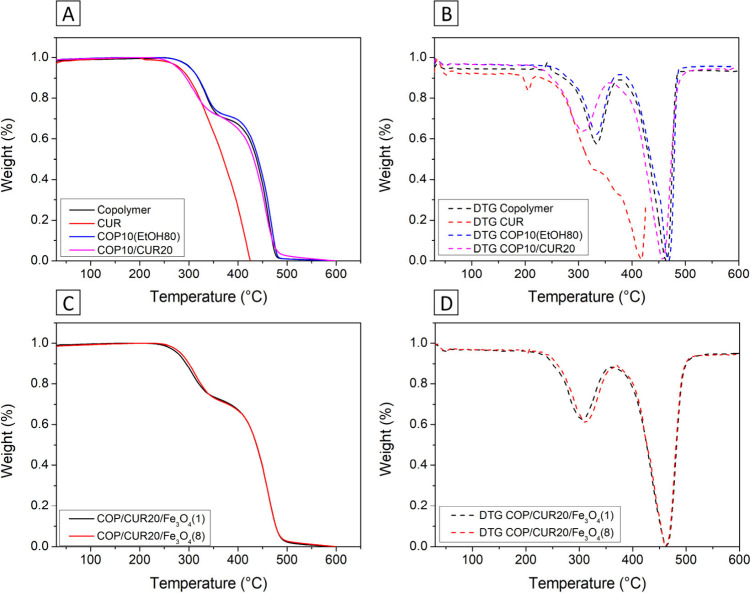
Thermogravimetric analysis of (A) P­(BMA-*co*-DMAEMA-*co*-MMA) copolymer, CUR, and microparticles COP10­(EtOH80) and COP10/CUR20: (A) TGA and (B) DTG curves; and composite microparticles COP/CUR20/Fe_3_O_4_(1) and COP/CUR20/Fe_3_O_4_(8): (C) TGA and (D) DTG curves.

The COP10/CUR20 sample showed a greater mass alteration due to incorporating 20% CUR into its composition. The initial mass-change temperature of CUR is approximately 200 °C, which is consistent with values reported in the literature.^55^ CUR’s TGA/DTG profile exhibits a single degradation event, with no indication of water evaporation due to its hydrophobic nature.^51^

The COP/CUR20/Fe_3_O_4_(1) and COP/CUR20/Fe_3_O_4_(8) exhibited greater thermal stability due to the presence of iron oxide. This effect is enhanced by the uniform distribution of Fe_3_O_4_ throughout the composite system, which delays the degradation process.^56^ The incorporation of Fe_3_O_4_ nanoparticles confers enhanced resistance to the material. Fe_3_O_4_ can interact with the tertiary amine groups of the DMAEMA units, forming polymer–particle junctions that act as physical/ionic cross-links and contribute to the formation of a polymer–particle network. The iron oxide also reinforces the structure of the microparticles.^57^

The WAXS profiles of CUR, P­(BMA-*co*-DMAEMA-*co*-MMA) copolymer, and the microparticles COP10­(EtOH80), COP10/CUR20, COP/CUR20/Fe_3_O_4_(1), and COP/CUR20/Fe_3_O_4_(8) are shown in Figure 8. The XRD pattern of CUR shows sharp, intense peaks in the 2θ range of 5° to 60°, characteristic of a crystalline material.^51^ In contrast, the COP10­(EtOH80) and the copolymer showed broader peaks with lower intensities, indicating their amorphous nature.^53^

**Figure 8:**
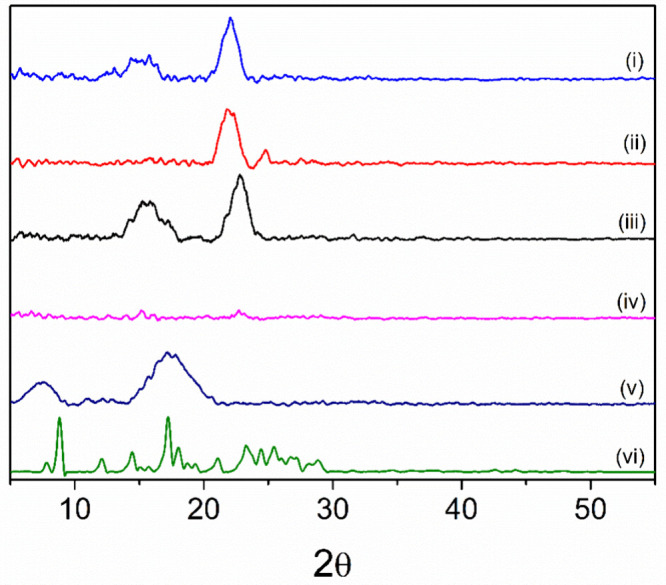
XRD profiles of (i) COP10/CUR20/Fe_3_O_4_(8), (ii) COP/CUR20/Fe_3_O_4_(1), (iii) COP10/CUR20, (iv) COP10­(EtOH80), (v) copolymer, and (vi) CUR.

The COP10/CUR20 and COP/CUR20/Fe_3_O_4_(8) exhibited diffraction peaks at 2θ = 15.2° and 22.8°, whereas COP/CUR20/Fe_3_O_4_(1) displayed peaks at 2θ = 22.8° and 24.8°. Neither COP/CUR20/Fe_3_O_4_(1) nor COP/CUR20/Fe_3_O_4_(8) showed characteristic peaks corresponding to the presence of Fe_3_O_4_, even in the sample with the highest incorporated concentration of 8% (w/w). This is likely attributed to the relatively low iron oxide concentration compared to the organic matrix of the composite material.

### 3.7 *In Vitro* CUR Release

To investigate the release mechanism, the percentage of CUR released from the microparticles was plotted as a function of time (Figure 9). Release assays were previously conducted for the COP10/CUR20 and pure CUR (control) in simulated intestinal fluid (SIF, pH 6.8) and in sodium acetate/acetic acid buffer (pH 3.8) at 37 °C (Figure 9).

**Figure 9:**
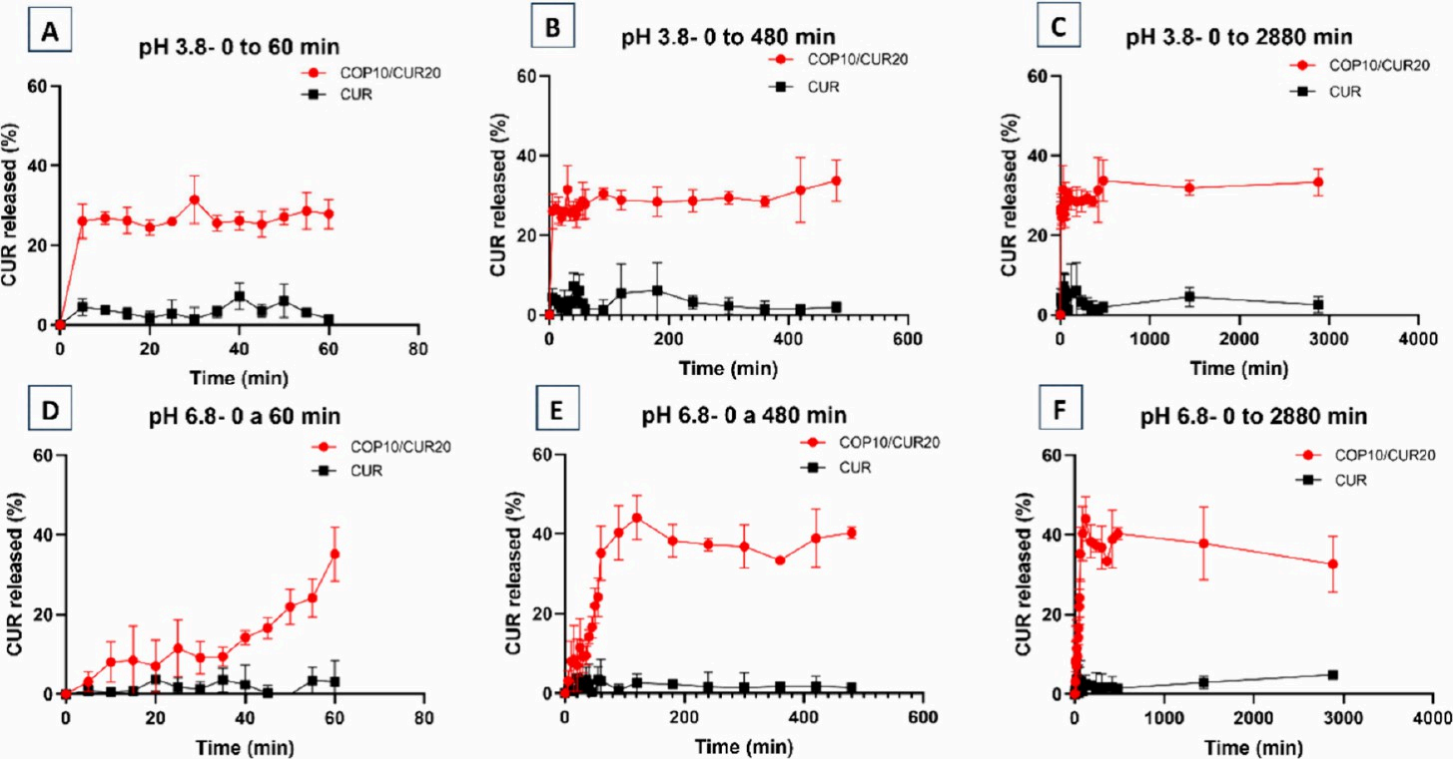
Release profile of encapsulated CUR (COP10/CUR20) and raw CUR control. The CUR control curve was obtained by adding pure CUR to the buffers and then measuring the absorbance of the free CUR present in the supernatant. Release assays at pH 3.8: (A) from 0 to 60 min; (B) from 0 to 480 min; (C) from 0 to 2880 min. Release assays at pH 6.8: (D) from 0 to 60 min; (E) from 0 to 480 min; (F) from 0 to 2880 min.

Pure CUR exhibited lower solubility than encapsulated CUR, which was released from the COP10/CUR20 microparticles. At pH 3.8, pure CUR reached a maximum solubility of 6.13% (±7) or 15.3 mg/g after 180 min (3 h). At pH 6.8, its solubility was 4.9% (±0.3) or 12 mg/g after 2880 min (48 h). In contrast, COP10/CUR20 microparticles released 33.3% (±3.3) or 83.25 mg/g of CUR after 480 min (8 h) at pH 3.8, and 44% (±5.5) or 110 mg/g after 120 min (2 h) at pH 6.8, demonstrating a rapid dissolution profile for the encapsulated drug. The release rate increased from 2.04%/h (pure CUR) to 4.16%/h (encapsulated CUR) at pH 3.8, and from 0.10%/h to 22%/h at pH 6.8. These results are attributed to the amorphous state of the encapsulated CUR, ensuring a superior dissolution and release profile in aqueous media.^58^

These results agree with other findings. Li et al.^1^ developed a copolymer/CUR solid dispersion via the solution mixing method to enhance CUR’s properties for biomedical applications. This solid dispersion increased CUR solubility in water to at least 3 mg/mL, improving its stability and bioavailability. The copolymer protected CUR against hydrolysis and prevented drug precipitation over a pH range of 5, 6, 7, and 8, as well as under UV irradiation, protecting both direct (50%) and indirect (85%) photolysis. *In vitro* transdermal permeation tests were also conducted, showing that the solid dispersion achieved a permeation rate of 16%.

Complementarily, Kerdsakundee et al.^59^ produced copolymer/CUR solid dispersions to prolong gastric residence time and promote the controlled release of CUR to treat gastric ulcers. The dispersions were prepared by solvent evaporation in different ratios to increase CUR solubility. The formulation with the highest solubility (3.92 mg/mL) used a 1:5 CUR/copolymer ratio. The optimal formulation also contained 1% sodium alginate, 0.5% calcium carbonate, and 1% sodium bicarbonate, which formed a gastric gel “raft” and released 85% of the drug within 8 h. Oral administration of CUR in this formulation, at a dose of 40 mg/kg once daily, demonstrated high efficacy in healing chronic gastric ulcers and reducing the dosing frequency compared to conventional CUR suspension.

To evaluate the CUR release behavior under the influence of an external magnetic field, the release rates of CUR from COP/CUR20/Fe_3_O_4_(1) (Figure 10) and COP/CUR20/Fe_3_O_4_(8) (Figure 11) were analyzed in the presence and absence of an external magnetic field.

**Figure 10:**
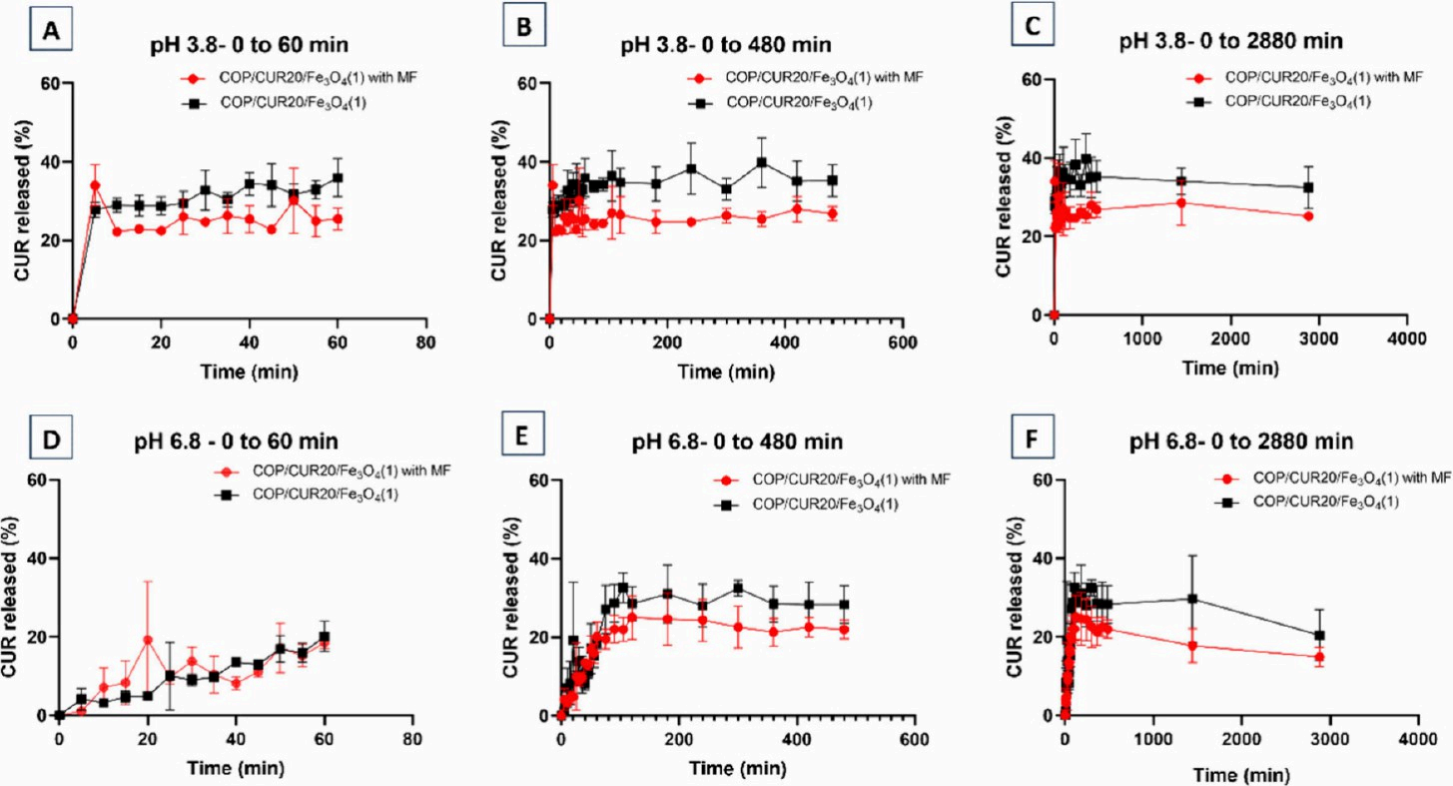
Release of CUR from COP10/CUR20/Fe_3_O_4_(1) samples with and without an external magnetic field (MF). Release assay at pH 3.8: (A) from 0 to 60 min; (B) from 0 to 480 min; (C) from 0 to 2880 min. Release assay at pH 6.8: (D) from 0 to 60 min; (E) from 0 to 480 min; (F) from 0 to 2880 min.

**Figure 11:**
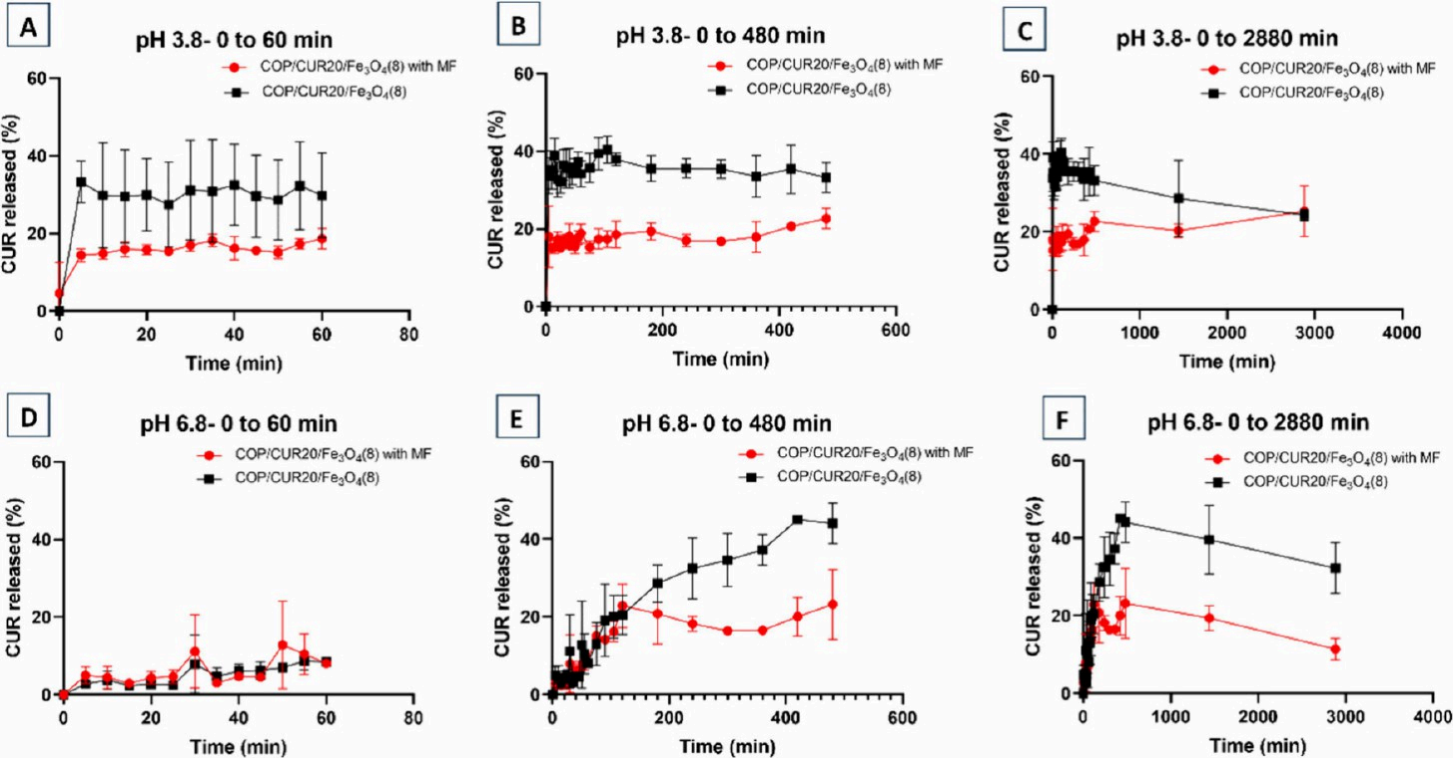
Release of CUR from COP10/CUR20/Fe_3_O_4_(8) with and without a magnetic field (MF). Release assay at pH 3.8: (A) from 0 to 60 min; (B) from 0 to 480 min; (C) from 0 to 2880 min. Release assay at pH 6.8: (D) from 0 to 60 min; (E) from 0 to 480 min; (F) from 0 to 2880 min.

In the acetate buffer, after 360 min (6 h), CUR release from the COP/CUR20/Fe_3_O_4_(1) reached its maximum percentage (40% ± 6.99 mg/g). After this period, a decrease in CUR concentration was observed, indicating its degradation, which dropped to 32% ± 5 (80 mg/g) after 2880 min (48 h). In SIF medium, CUR release from the COP/CUR20/Fe_3_O_4_(1) achieved 32% ± 2 (80 mg/g) at 300 min (5 h) and decreased to 20% ± 6 (50 mg/g) after 48 h.

For the COP/CUR20/Fe_3_O_4_(8) in sodium acetate/acetic acid buffer, the maximum release occurred at 105 min (1 h 45 min), reaching 40% ± 3 (99 mg/g). After 48 h, the release decreased to 24% ± 1 (60 mg/g) (Figure 11). Notably, the CUR release remained virtually constant from the first 5 min of analysis. P­(BMA-*co*-DMAEMA-*co*-MMA) copolymer used for CUR encapsulation, contains tertiary amine groups that become ionized in acidic media, making it highly soluble at pH values below 5.0.^60^ Thus, with the complete solubilization of the copolymer, instantaneous CUR release occurred.

In SIF medium (pH 6.8), the COP/CUR20/Fe_3_O_4_(8) released CUR with a maximum percentage of 45% ± 1 (112.5 mg/g) at 420 min (7 h), stabilizing at 32% ± 6 (80 mg/g) after 48 h. In this case, the release occurred rapidly but continued progressively over time. Thus, for COP/CUR20/Fe_3_O_4_(1) and COP/CUR20/Fe_3_O_4_(8) samples subjected to the external magnetic field, CUR release in acetate buffer was 25% ± 7 (62.5 mg/g) after 48 h for COP/CUR20/Fe_3_O_4_(1) and 28% ± 6 (70 mg/g) after 1440 min (24 h) for COP/CUR20/Fe_3_O_4_(8). After 48 h, the COP/CUR20/Fe_3_O_4_(8) showed a decline in CUR release, reaching 25% ± 1 (62.5 mg/g). In SIF, the COP/CUR20/Fe_3_O_4_(1) released 25% ± 6 (62.5 mg/g) of CUR at 120 min (2 h), decreasing to 22% ± 1 (55 mg/g) after 48 h, whereas the COP/CUR20/Fe_3_O_4_(8) released 23% ± 9 (57.5 mg/g) after 48 h. An external magnetic field may promote the aggregation of magnetic particles, thereby increasing the diffusion path length (tortuosity) and delaying release.^61^

Among the highest release rates, the application of a magnetic field reduced the CUR release rate. For the COP/CUR20/Fe_3_O_4_(1), the release rate decreased from 6.6%/h to 0.52%/h at pH 3.8. The COP/CUR20/Fe_3_O_4_(8) showed even more promising results, with a reduction from 27.5%/h to 1.16%/h at pH 3.8 and 6.42%/h to 0.48%/h at pH 6.8.

These findings agree with other studies. In their study, Liu et al.^17^ confirmed the influence of the magnetic field on the release process using a superparamagnetic Fe_3_O_4_–MoO_4_ nanocomposite. In this study, the release rate was approximately 27.25%/h; upon applying a magnetic field, it was reduced to 4.42%/h. When the magnetic field was removed, the release rate increased again to 9.43%/h; however, reapplication of the magnetic field led to a further reduction. This behavior suggests that release control can be effectively achieved by applying an external magnetic field.

Almeida et al. developed pH- and temperature-responsive magnetic microparticles using pectin maleate, N-isopropylacrylamide, and Fe_3_O_4_ nanoparticles.^49^ The aim was to apply these microparticles for the controlled release of CUR in SGF and SIF under different temperature conditions (25 or 37 °C). The Fe_3_O_4_-loaded microparticles exhibited slow CUR release in the presence of a magnetic field. Additionally, encapsulated CUR demonstrated improved stability and greater solubility than free CUR. At 25 °C in SIF, without magnetic-field influence, the release equilibrium was reached in 35 h, with 50% CUR released. Under the same conditions, equilibrium was reached in 80 h, with 90% CUR released in the presence of the magnetic field. In SGF at 25 °C, the release rate did not exceed 10% under any of the tested conditions. At 37 °C, the magnetic field did not significantly alter the CUR release profile. In SIF (37 °C), the released fraction reached 95% and 80% in the presence and absence of a magnetic field, respectively. The released CUR content was lower in SGF (37 °C), with 20% of it being released without and 6% with magnetic field influence.

### 3.8 Transport Mechanism

The CUR release profiles from the electrosprayed P­(BMA-*co*-DMAEMA-*co*-MMA) macroparticles were determined by regression analysis of experimental data using the zero-order, pseudo-first-order, Higuchi, and Korsmeyer–Peppas kinetic models. The best fit for the release data was determined by comparing the correlation coefficients (*R* ^2^) of each model. Figure 12 shows the kinetic curves for CUR release from COP/CUR20/Fe_3_O_4_(8) at pH 3.8 and 6.8.

**Figure 12:**
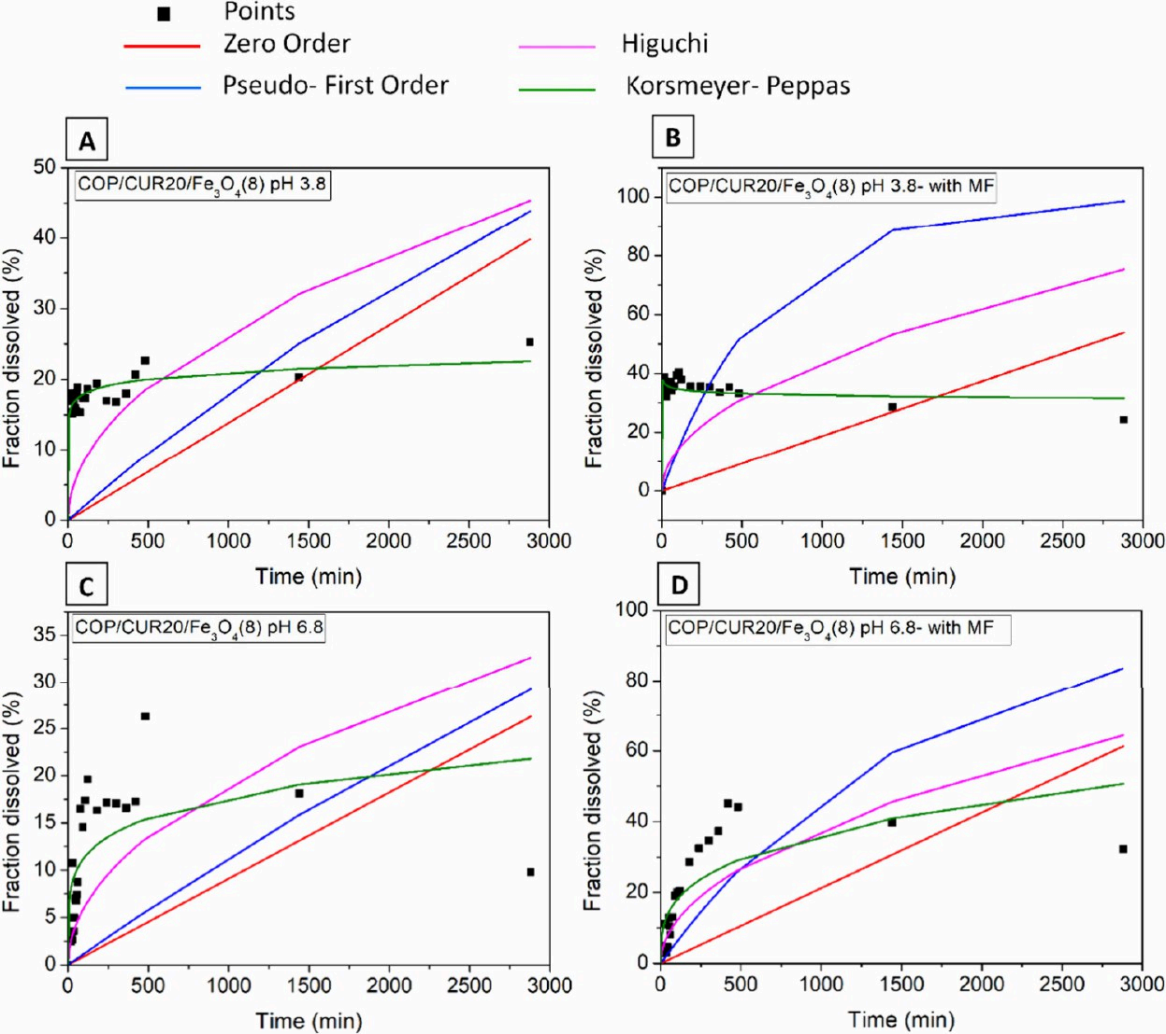
CUR release curves from COP/CUR20/Fe_3_O_4_(8) obtained at pH 3.8 and 6.8 fitted with kinetic models, including nonlinear zero-order, pseudo-first-order, Higuchi, and Korsmeyer–Peppas. (A and C) without magnetic field (MF) application, (B and D) with magnetic field (MF) application.

The Korsmeyer–Peppas model provided the best fit to the release kinetic data for CUR from COP10/CUR20, COP/CUR20/Fe_3_O_4_(1), and COP/CUR20/Fe_3_O_4_(8). This result was obtained at both pH 3.8 and pH 6.8, in the presence and absence of an external magnetic field (Figure 12). The parameters obtained from the kinetic curve fitting using the Korsmeyer–Peppas model are presented in Table 4.

**Table 4:** Kinetic Parameters (*n*, *K*_kp_, *R* ^2^) Obtained by Applying the Korsmeyer–Peppas Mathematical Model in the CUR Release Data Obtained in Acetate Buffer (pH 3.8) and SIF (pH 6.8) at 37 °C^a^

	Sample	*n*	*K*_kp_	*R* ^2^
pH 3.8	COP10/CUR20	0.045	23.289	0.9673
	COP/CUR20/Fe_3_O_4_(1)	0.035	28.395	0.9457
	COP/CUR20/Fe_3_O_4_(1) with MF	0.003	25.40	0.9457
	COP/CUR20/Fe_3_O_4_(8)	0.067	13.162	0.9313
	COP/CUR20/Fe_3_O_4_(8) with MF	–0.028	39.473	0.9255
pH 6.8	COP10/CUR20	0.208	9.963	0.779
	COP/CUR20/Fe_3_O_4_(1)	0.169	9.272	0.7247
	COP/CUR20/Fe_3_O_4_(1) with MF	0.143	8.322	0.6907
	COP/CUR20/Fe_3_O_4_(8)	0.195	4.618	0.6816
	COP/CUR20/Fe_3_O_4_(8) with MF	0.310	4.294	0.8237

a MF = Magnetic field.

The Korsmeyer–Peppas model, described by the semiempirical equation (eq 1), is based on Fickian diffusion and accounts for the release of hydrophobic molecules from hydrophilic matrixes.^62^

1 $$\frac{M_{t}}{M_{\infty }}=K_{kp}t^{n}$$

The term *M*_*t*_/*M*_∞_ refers to the fraction of drug released at a given time (*t*); *M*_*t*_ is the amount of drug released at time *t*; *M*_∞_ is the amount of drug released at time ∞; *n* is the diffusional exponent or drug release exponent; and *K*_kp_ is the Korsmeyer release constant.^58^

The diffusional exponent (*n*) can be used to characterize different release profiles in polymeric matrixes and to describe the drug release mechanism.^63^ When *n* is less than 0.5, the release mechanism is predominantly governed by Fickian diffusion.^64^ For an *n* value equal to 0.89, the release is described by a zero-order kinetic, indicating a linear increase in the solute released over time. Values between 0.5 and 0.89 suggest anomalous transport, characterized by a combination of diffusional mechanisms and relaxation of the polymer matrix.^33^ In the context of the release kinetics performed with the P­(BMA-*co*-DMAEMA-*co*-MMA) microparticles, the observed *n* value indicates that Fickian diffusion prevails in CUR release, with *n* being less than 0.5.

## 4 Conclusions

In this work, the electrospraying parameters were systematically optimized for P­(BMA-*co*-DMAEMA-*co*-MMA) copolymer solutions with and without CUR and iron oxide, enabling the production of composite microparticles with well-defined morphology. The poly­(butylmethacrylate-*co*-(2-dimethylaminoethyl) methacrylate-*co*-methyl methacrylate) copolymer is a widely used thermoplastic methacrylate copolymer that is recyclable. It has been employed as a protective matrix and drug-encapsulation vehicle due to its favorable biocompatibility and capacity to form stable particles.

A stable electrospraying process was obtained for copolymer concentrations below 25% (w/v), and the resulting microparticles effectively encapsulated CUR, indicating significant potential for enhancing its apparent solubility in aqueous media. FTIR-ATR and XRD analyses confirmed the incorporation of CUR into the amorphous or partially amorphous polymeric matrix. The presence of Fe_3_O_4_ in the microparticles was verified by FTIR-ATR and thermal analyses (DSC and TGA), and the incorporation of iron oxide improved the thermal stability and reduced the degradation rate of the composite microparticles.

CUR release studies revealed that the application of an external magnetic field markedly influenced the drug release rate under both acidic and near-neutral pH conditions. The COP10/CUR20/Fe_3_O_4_(8) formulation showed the most promising performance, reducing the release rate of CUR from 27.5%/h to 1.16%/h at pH 3.8 and from 6.42%/h to 0.48%/h at pH 6.8 when exposed to a magnetic field. Kinetic analysis indicated that the Korsmeyer–Peppas model best fitted the experimental data, suggesting that CUR release is predominantly governed by Fickian diffusion of hydrophobic molecules through a hydrated, pH-responsive matrix. The combined effects of matrix ionization and the increased tortuosity associated with Fe_3_O_4_ incorporation and magnetic field application contribute to the substantial reduction of the initial burst release.

Compared with previously reported CUR delivery systems based on poly­(methacrylate) matrixes or magnetic composites, the present study demonstrates that electrosprayed P­(BMA-*co*-DMAEMA-*co*-MMA)/Fe_3_O_4_ microparticles can simultaneously achieve high CUR loading, reduced crystallinity, dual (pH- and magnetically) responsive release, and significant suppression of burst release. This dual-stimuli behavior represents a relevant advancement over systems that rely solely on pH sensitivity or passive diffusion control.

This study has some limitations. Scaling up the electrospraying process to industrially relevant throughputs remains challenging and will require further engineering optimization. In addition, the minimum CUR concentration required to ensure antimicrobial or therapeutic efficacy was not established and should be assessed in future *in vitro* and *in vivo* studies. Ensuring homogeneous dispersion of Fe_3_O_4_ nanoparticles within the microparticles is also critical, as local aggregation may affect both magnetic responsiveness and release profiles. Another important aspect is CUR’s high sensitivity to external conditions (light exposure, pH, and temperature), which may lead to degradation during processing or storage and should be systematically investigated.

Overall, the results confirm the strong potential of these magnetic, pH-responsive composite microparticles to enhance CUR solubility and enable its controlled release. In particular, the ability to modulate CUR release by combining pH responsiveness with external magnetic fields suggests that these systems may be further explored as advanced platforms for site-specific, on-demand drug delivery. Potential application areas include wound healing, local infection control, and other biomedical settings where localized, stimulus-responsive CUR delivery is desired.
